# Does Attentional Selectivity in the Flanker Task Improve Discretely or Gradually?

**DOI:** 10.3389/fpsyg.2012.00434

**Published:** 2012-10-26

**Authors:** Ronald Hübner, Lisa Töbel

**Affiliations:** ^1^Fachbereich Psychologie, Universität KonstanzKonstanz, Germany

**Keywords:** selective attention, continuous versus discrete, flanker task, modeling, diffusion models

## Abstract

An important question is whether attentional selectivity improves discretely or continuously during stimulus processing. In a recent study, Hübner et al. (2010) found that the discrete Dual-Stage Two-Phase (DSTP) model accounted better for flanker-task data than various continuous-improvement models. However, in a subsequent study, White et al. (2011) introduced the continuous shrinking-spotlight (SSP) model and showed that it was superior to the DSTP model. From this result they concluded that attentional selectivity improves continuously rather than discretely. Because different stimuli and procedures were used in these two studies, though, we questioned that the superiority of the SSP model holds generally. Therefore, we fit the SSP model to Hübner et al.’s data and found that the DSTP model was again superior. A series of four experiments revealed that model superiority depends on the response-stimulus interval. Together, our results demonstrate that methodological details can be crucial for model selection, and that further comparisons between the models are needed before it can be decided whether attentional selectivity improves continuously or discretely.

## Introduction

Selective spatial attention is an important control mechanism for goal-directed behavior. Accordingly, it has intensively been investigated during the last decades. One idea of how specific information is selected from the visual field is to assume some kind of spatial attentional filtering. For instance, based on results obtained with the spatial-cueing paradigm, some researchers proposed that such filtering proceeds like an *attentional spotlight*, i.e., that visual attention can be allocated to a certain location and that items at that location are processed more intensively than items at other locations (Posner, 1980; Posner et al., 1980). Further important properties of spatial attention have also been revealed by the flanker task (Eriksen and Eriksen, 1974), in which participants have to categorize a target stimulus as fast and as accurately as possible, while ignoring irrelevant flanker stimuli. The flankers are usually congruent, i.e., associated with the same response as the target, or incongruent, i.e., associated with the opposite response. The degree to which the flankers can be ignored or filtered out is assessed by the difference between the performance for congruent and incongruent stimuli, which is called the *flanker congruency effect*. Usually, responses to congruent stimuli are faster and more reliable than responses to incongruent flankers and the size of differences in RT and error rate (ER) are considered as measures of the efficiency of selective attention. Results obtained with the flanker task have led to the *attentional zoom-lens* metaphor, which generalizes the spotlight idea by not only assuming a variable position of the attentional filter, but also a variable size and form (Eriksen and Schultz, 1979; Eriksen and St James, 1986).

The regularly observed flanker congruency effect clearly indicates that selectivity is limited. Moreover, Gratton et al. (1988) analyzed distributional data and found that this limit changes in time. Usually, accuracy on incongruent trials is much higher for slow than for fast responses, indicating that attentional selectivity improves during the course of processing. In view of such results it has been hypothesized that stimulus processing is unselective in a first phase of processing, but then, after some time, enters a second phase with relatively high selectivity (e.g., Gratton et al., 1992). In more recent models, it has also been assumed that the increase in selectivity is controlled by some conflict monitoring mechanism. Accordingly, attentional selectivity is increased only after a response conflict is detected, which also leads to an unselective and a selective phase, at least for incongruent stimuli (e.g., Davelaar, 2008; Yu et al., 2009). Yet, as the zoom-lens metaphor already suggests, a discrete and stage-like improvement of selectivity is not the only way to account for the dynamics of selective attention. It is also possible that selectivity increases continuously with processing time by a gradually narrowing attentional focus on the target item (e.g., Heitz and Engle, 2007). Because the continuous account seems plausible and is relatively easy to formalize, it has been implemented in the frameworks of neural-networks (e.g., Cohen et al., 1992; Liu et al., 2008), of Bayesian observers (e.g., Yu et al., 2009), and of diffusion processes (e.g., Liu et al., 2009).

**Figure 1:**
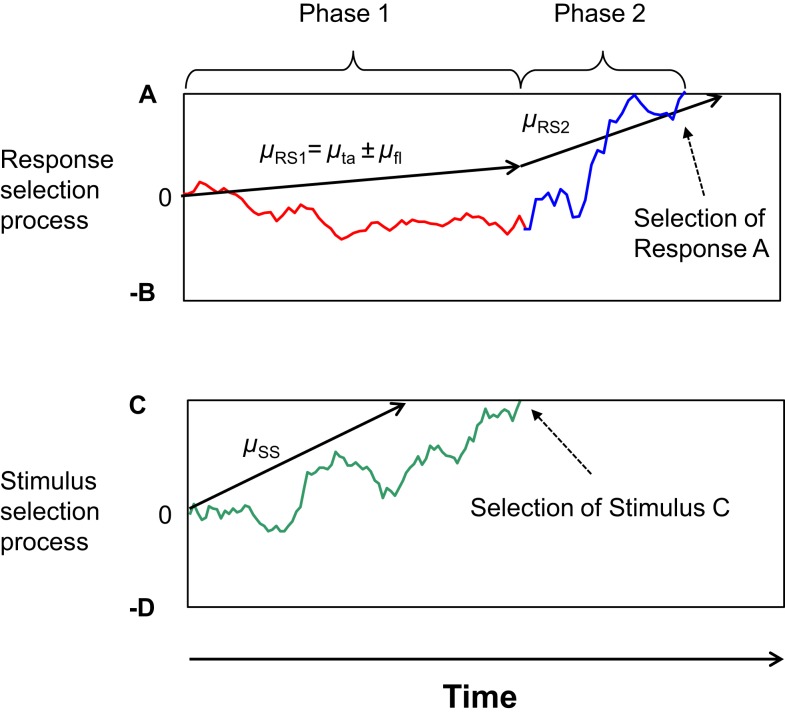
**Outline of the two phases of response selection in the DSTP model**. The upper graph represents the response-selection process, whereas the lower graph depicts the stimulus-selection process. In this example stimulus selection (late selection) is successful and selects the correct stimulus. Because response selection has not finished yet at that time, the stimulus selection has the effect that the rate of evidence accumulation for response selection increases, which defines the beginning of Phase 2 of response selection. The slope of the arrows represents the respective rate. The trajectories represent examples of single sample paths.

Recently, however, the idea of a discrete and stage-like improvement of selectivity has also been formalized by Hübner et al. (2010). Their Dual-Stage Two-Phase (DSTP) model relies on the assumption of two discrete stages of stimulus selection, an early stage of low selectivity and a late stage of high selectivity. The information provided by these two stages drives response selection in a first and second phase, respectively. Both phases are modeled by a diffusion process (cf. Ratcliff, 1978). Such processes are basically characterized by a drift rate reflecting the evidence available for responses A and B, and two corresponding thresholds *A* and −*B*. Hübner et al. (2010) assumed that in the first phase of response selection the rate is simply the sum of two component rates μ_ta_ and μ_fl_ for target and flankers, respectively. If the flankers are incompatible, then μ_fl_ is negative, which reduces the overall rate. Because the magnitudes of these component rates are modulated by attentional weights (attentional filtering), this part of the model represents *early* selection. Additionally, though, a *late* stimulus-selection process runs in parallel with response selection. It is also implemented as a diffusion process with drift rate μ_SS_, and selects the target or flanker depending on whether the accumulated evidence first reaches threshold *C* or −*D*, respectively. If the target is selected before a response, then the rate of response selection increases to a value μ_RS2_, which accounts for the improved accuracy of slower responses. It should be noted that the DSTP model accounts for the dynamics of selectivity within a trial without the assumption of conflict monitoring. An outline of the model with an example process is shown in Figure 1.

Hübner et al. (2010) compared the DSTP model to several continuous-improvement models, including the neural-network model of Cohen et al. (1992), which was also implemented as a diffusion-process models (Liu et al., 2008). Accordingly, the improvement of attentional selectivity in the alternative models was generally realized by a continuously increasing drift rate for response selection. However, the function of how the rate increased with time differed between the models. Fitting the different models to various distributional flanker-task data revealed that the DSTP model was superior, suggesting that attentional selectivity improves discretely rather than continuously. However, a general problem is that there are an infinite number of ways of how selectivity can increase continuously in time, so that Hübner et al. (2010) may simply not have found an optimal member of this model class. Indeed, White et al. (2011) questioned that the assumption of discrete selectivity generally explains data better than continuous selectivity, and proposed a specific *shrinking-spotlight* (SSP) model, also implemented as a diffusion process.

In the SSP model the overall rate for a given stimulus is also computed from the weighted evidence provided by each sub-component or item. It is assumed that all items provide the same amount of perceptual evidence *p*. However, the attentional weight for each item is determined by the proportion of the “spotlight” that falls on the item’s location in the display. Selectivity, and consequently the drift rate for incongruent stimuli, increases gradually as the width of the target-centered spotlight shrinks over time at a linear rate, *r*_d_, from *sd*_0_ to a minimum.

White et al. (2011) applied the SSP model together with discrete selection models, including a simplified version of the DSTP model, to flanker-task data and found that their model was superior. Based on this result, they concluded that processing in the flanker task is better described by gradual than by discrete attentional narrowing, which is contrary to the conclusion of Hübner et al. (2010). Therefore, from our perspective, the crucial question was whether the superiority of the SSP model to the DSTP model holds generally. Because White et al. used a different experimental method than Hübner et al. (2010), it was possible that the SSP model is superior to the DSTP model only under specific conditions.

It is certainly impossible to compare the two models under all possible methodological conditions. However, if selectivity improves continuously, as proposed by White et al. (2011) then one would expect that the SSP is at least also superior in accounting for Hübner et al.’s (2010) flanker-task data. Therefore, in a first step we implemented the SSP model and fit it to the distributional data from the three experiments (eight conditions) of Hübner et al.’s (2010) study using the same fitting procedure as in that study (for details see also below). The obtained goodness-of-fit measures are shown in Table 1. For comparison, not only the values for the SSP model are listed, but also those for the DSTP model and the best fitting continuous model from Hübner et al. (2010). The latter model has a non-linearly increasing rate and can be considered as equivalent to the neural-network model of Cohen et al. (1992).

**Table 1:** **Fit statistics of different models for the three experiments and corresponding conditions in Hübner et al. (2010)**.

Experiment/condition	Model	*G^2^*	*df*	BIC
Experiment 1:	DSTP	12.4	15	59.9
*Wide*	Non-linear increase	26.6	13	81.2
	SSP	48.5	17	82.4
Experiment 1:	DSTP	15.7	15	63.2
*Narrow*	Non-linear increase	42.3	13	103
	SSP	56.2	17	90.1
Experiment 2:	DSTP	7.86	15	50.3
*1-position-central*	Non-linear increase	18.8	13	73.3
	SSP	24.7	17	55.0
Experiment 2:	DSTP	10.0	15	52.5
*2-positions-lateral*	Non-linear increase	25.5	13	80.0
	SSP	26.6	17	56.9
Experiment 2:	DSTP	8.18	15	47.7
*3-positions-central*	Non-linear increase	19.2	13	70.0
	SSP	15.4	17	43.7
Experiment 2:	DSTP	17.7	15	62.2
*3-positions-lateral*	Non-linear increase	40.7	13	97.9
	SSP	34.0	17	65.9
Experiment 3:	DSTP	12.6	15	61.6
*20%-congruent*	Non-linear increase	25.7	13	89.7
	SSP	26.0	17	61.0
Experiment 3:	DSTP	25.3	15	74.3
*80%-congruent*	Non-linear increase	51.1	13	114
	SSP	60.6	17	95.6

*In Experiment 1 the spacing (wide, narrow) between target and flanker was varied, in Experiment 2 the stimulus position and spatial uncertainty, and in Experiment 3 the proportion of congruent versus incongruent stimuli. The values for the DSTP model and the non-linear increase model are reproduced from Hübner et al. (2010), whereas the values for the SSP model are obtained by new fits. *G*^2^, Wilks likelihood ratio chi-square; *df*, degrees of freedom; BIC, Bayesian information criterion*.

As can be seen in Table 1, with respect to the *G*^2^ (Wilks likelihood ratio chi-square) values, which represent a basic measure of fit (cf. Ratcliff and Smith, 2004), the DSTP model is superior for the data in all experiments and conditions. The table also shows the Bayesian information criterion (BIC) model-selection statistics (Schwarz, 1978), which takes the number of model parameters into account. According to this statistic, the model with the smaller BIC should be preferred. If we consider these values in Table 1, then we see that the BIC for the SSP model is slightly smaller (61.0 versus 61.6) than that for the DSTP model only in the *20%-congruent* condition. In all other conditions, though, the DSTP model still yields better results. Compared to the non-linear increase model, the BIC for the SSP model was always superior except for the *wide* condition. Thus, the SSP model is a parsimonious model that, in terms of BIC, is more successful than the best continuous-improvement model considered in Hübner et al. (2010). However, it is still less successful compared to the DSTP model.

These comparisons show that the SSP model is not generally superior to the DSTP model. Accordingly, the conclusion that attentional selectivity improves continuously is no longer justified. Rather, with respect to the different models, it is obvious that their superiority depends on methodological details. The method applied in Hübner et al. (2010) seems to be favorable for the DSTP model, whereas that in White et al. (2011) is advantageous for the SSP model.

Thus, a further aim of the present study was to investigate which details of the applied methods are crucial for model superiority. Of the differences between the studies those with respect to stimuli and tasks were most striking. Although both studies used a flanker task, Hübner et al. required parity judgments on numerals, whereas White et al.’s (2011) participants had to indicate the pointing direction of arrows. Therefore, after verifying in our first experiment that White et al.’s (2011) result was replicable in our lab, we combined in Experiment 2 the arrow stimuli and the corresponding task with the procedure in Hübner et al. (2010). As a result, the DSTP model was now better than the SSP model, which indicated that some other variable must be crucial for model superiority. The experiments still differed in stimulus duration, response-stimulus interval (RSI), error feedback, and responding. Because there were striking differences in the variance of the latencies between the experiments, we speculated that stimulus duration might be an important factor. However, its variation in Experiment 3 had no effect on model superiority. Therefore, we next examined the effect of the RSI, because this factor is known to affect automatic as well as controlled process (e.g., Soetens et al., 1985). Indeed, the result of Experiment 4, combined with that of Experiment 3, shows that a relatively long RSI leads to data that are fit better by the DSTP than by the SSP model, whereas the opposite holds for a relatively short RSI.

## Experiment 1

In our first experiment we tried to replicate White et al.’s (2011) results. To this end, we collected data by applying the same stimuli and procedure as in that study. Specifically, we used vertically arranged arrows as stimuli and a “left” or “right” decision as task, and also adopted the other procedural details from White et al. (2011). If the task and procedure matter for model superiority, then the SSP model should again fit the data better than the DSTP model.

### Method

#### Participants

Eighteen participants (mean age 24.4 years, five male) with normal or corrected-to-normal vision, participated in the study. They were recruited at the Universität Konstanz and were paid 8 €/h.

#### Apparatus and stimuli

Stimuli were presented on a 19″-monitor with a resolution of 1280 × 1024 pixels, and a personal computer (PC) served for controlling stimulus presentation and response registration. The item set was the same as in White et al. (2011) and consisted of left or right pointing arrows (<, >). Participants were seated at a distance of about 45 cm from the screen, so that the width and height of the arrows subtended a visual angle of approximately 0.7°. Stimuli were presented in white on a black background. The target arrow always appeared at the center of the screen. Flanker arrows (two above, and two below the target) were arranged vertically as in White et al. (2011). The separation between the items was always 0.4°. For congruent stimuli, the flanker arrows pointed in the same direction as the target arrow, whereas for incongruent stimuli the flankers pointed in the opposite direction.

#### Procedure

Stimuli were presented at the center of the screen and remained on the display until response. The task was to decide whether the target arrow pointed to the left or to the right, and to indicate the decision by pressing corresponding keys “y” and “–” on the keyboard (German layout) with their index finger of their left and right hand, respectively. Stimuli were congruent on half of the trials and incongruent on the other half. One second after the response, the next trial began. No error feedback was given. After an RSI of 350 ms the next stimulus appeared.

Participants first performed a 48-trials practice block, and then worked through 16 test blocks of 64 trials each in a 45 min session. Outliers were controlled by eliminating the fastest and slowest responses. Cut-offs were chosen in such a way that less than 1% of the data were excluded (cf. Ulrich and Miller, 1994). For the present experiment this means that responses faster than 250 ms or slower than 1500 ms were excluded from analysis (<0.9% of the data).

#### Model fitting

To examine model performance, responses-time distributions for correct and incorrect responses in each condition (congruent, incongruent) were constructed by quantile-averaging (0.1, 0.3, 0.5, 0.7, and 9) the data. By this procedure, the data of each condition were sorted into six bins comprising 10, 20, 20, 20, 20, and 10% of the data, respectively. One exception were the error responses for congruent stimuli. Because they occurred rarely, only the 0.5 quantile was used for representing the corresponding RTs, as in White et al. (2011), which produced only two bins (50, 50%). Computer-simulation versions of the DSTP and the SSP model were then fit to these distributions with the same fit procedure as in Hübner et al. (2010). Specifically, the PRAXIS algorithm (Brent, 1973; Gegenfurtner, 1992) was applied to find parameter values for a given model that minimized the *G*^2^ statistics (cf. Ratcliff and Smith, 2004):

$$G^{2}=2\overset{J}{\underset{i=1}{\sum }}Np_{i}1\text{n}\left(\frac{p_{i}}{\pi_{i}}\right),$$

In this equation *J* is the number of bins, *p*_i_ is the proportion of observations in the *i*^th^ bin, and π_i_ is the proportion in this bin predicted by the considered model. *N* is the number of all observations^1^. Because the congruent and incongruent conditions were fit together, we had *J *= 20 bins (six for correct responses in the congruent condition, two for errors in the congruent condition, six for correct responses in the incongruent condition, and six for errors in the incongruent conditions).

Assuming symmetric thresholds (*A *= *B*, *C *= *D*), there were seven parameters for the DSTP model, including one parameter (*t*_er_) for representing the non-decisional time. The SSP model had five parameters. Let *J*_c_ and *J*_i_ be the number of bins for the congruent and incongruent condition, respectively, and *M* the number of model parameters, then the degrees of freedom (df) are calculated by df = (*J*_c_ − 1) + (*J*_i_ − 1) − *M*.

We simulated 8 × 10^5^ trials for each condition and fit cycle. To prevent that the obtained parameter estimates represent a local minimum, the fit procedure was repeated several times with different sets of initial parameter values.

### Results and discussion

#### Mean performance

The latencies of correct responses were analyzed by a one-factor ANOVA for repeated measures on the factor *congruency* (congruent, or incongruent). The analysis revealed a significant *congruency* effect, *F*(1, 18) = 126, *p *< 0.001. Responses were faster for congruent than for incongruent stimuli (Table 2). The mean ER was 7.28%. The ERs were subjected to an ANOVA of the same type as for the RTs. It revealed a significant effect of *congruency*, *F*(1, 18) = 42.2, *p *< 0.001, indicating that congruent stimuli produced a smaller ER than incongruent ones (Table 2).

**Table 2:** **Mean response times and their SD for correct responses, mean response times for error responses, and mean error rates for the different conditions in the four experiments**.

Experiment and condition	Mean correct RT	SD correct RT	Mean error rate	Mean error RT
**1**.				
Congruent	455 (62)	100 (29)	4.39 (2.57)	420 (71)
Incongruent	493 (64)	114 (29)	10.16 (5.37)	421 (58)
**2. HORIZONTAL**				
Congruent	374 (37)	61 (21)	2.18 (1.65)	365 (93)
Incongruent	443 (47)	88 (35)	14.13 (4.33)	359 (52)
**2. VERTICAL**				
Congruent	385 (37)	67 (28)	2.10 (1.68)	379 (70)
Incongruent	448 (46)	87 (25)	15.11 (3.87)	372 (42)
**3**.				
Congruent	379 (32)	72 (18)	1.19 (1.18)	377 (94)
Incongruent	426 (43)	86 (14)	8.32 (5.05)	363 (47)
**4**.				
Congruent	425 (47)	100 (31)	1.90 (1.11)	362 (58)
Incongruent	457 (51)	111 (33)	4.88 (2.17)	380 (45)

*SD across participants are shown in parenthesis*.

These results show the same pattern as those in White et al.’s (2011) first experiment. However, the responses in the present experiment were numerically faster (474 versus 505 ms), and the congruency effect was smaller in RT (Δ38 versus Δ78 ms) as well as in ER (Δ5.81 versus Δ7.6%).

#### Model fits

The parameters and goodness-of-fit values obtained from fitting the DSTP and the SSP model to the distributional data are also shown in Table 3. The table also shows BIC model-selection values (Schwarz, 1978), which also represent goodness-of-fit but additionally take the number of model parameters into account. Accordingly, the model with the smaller BIC should be preferred. As can be seen, although the pure goodness-of-fit (*G*^2^) was slightly better for the DSTP model, the BIC value is in favor (i.e., smaller) of the SSP model due to the fewer parameters of that model.

**Table 3:** **Parameter estimates and goodness-of-fit measures obtained by fitting the DSTP model and the SSP model to quantile-averaged response-time distributions for the different congruent and incongruent conditions**.

	Parameters									
**DSTP**										
***Exp./Cond***.	**μ_ta_**	**μ_fl_**	***A*/*B***	**μ_SS_**	**C/D**	**μ_RS2_**	***t*_er_**	***G^2^***	***df***	**BIC**

1.	0.0874	0.0705	0.0749	0.4437	0.1129	1.9799	0.2283	58.9	11	110
2. Hori	0.1067	0.1580	0.0705	0.5308	0.1034	1.3876	0.2034	26.4	11	70.8
2. Verti	0.0756	0.1483	0.0729	0.5343	0.1047	1.4589	0.2028	31.6	11	75.9
3.	0.1154	0.1423	0.0796	0.4885	0.0955	1.7074	0.1914	79.3	11	131
4.	0.0992	0.0570	0.0888	0.4219	0.1100	1.9426	0.1685	47.6	11	98.8
**SSP**										
***Exp./Cond***.		***p***	***A*/*B***	**r_d_**	**sd_a_**		***t*_er_**	***G^2^***	***df***	**BIC**

1.		0.2873	0.0540	0.0406	1.9650		0.2890	69.1	13	106
2. Hori		0.3944	0.0522	0.0234	1.9243		0.2504	53.5	13	85.2
2. Verti		0.3485	0.0528	0.0260	1.9267		0.2540	65.3	13	97.0
3.		0.3763	0.0527	0.0394	1.8290		0.2494	105	13	141
4.		0.3289	0.0598	0.0378	1.6645		0.2504	50.6	13	87.2

*Hori, horizontal stimulus arrangement; Verti, vertical-stimulus arrangement; *A/B*, response-selection boundaries; *t*_er_, mean non-decision time (in seconds); *p*, perceptual input; *s*d_a_, spotlight width; *r*_d_, rate of decrease in spotlight; *C/D*, stimulus-selection boundaries; μ_SS_, drift rate for stimulus selection; μ_RS2_, drift rate for response-selection phase 2; μ_ta_, drift rate from target; μ_fl_, combined drift rate for flankers; *G*^2^, Wilks likelihood ratio chi-square; *df*, degrees of freedom; BIC, Bayesian information criterion*.

Thus, by applying the task and procedure of White et al. (2011), and by fitting the models to the data we have to conclude that the SSP model is indeed superior to the DSTP model, at least under these specific conditions. The fact that the DSTP model is superior under other experimental conditions suggests that procedural differences produced the inconclusive results with respect to model superiority. The question now was which methodological details were responsible for the advantage of the SSP model in the present experiment. To answer this question, we conducted further experiments.

## Experiment 2

In this experiment we examined the role of stimulus type and task for model superiority. The hypothesis was that data obtained with arrow stimuli and the corresponding task might generally be better accounted for by the SSP model. If this is the case, then this model should also be superior to the DSTP model when arrow stimuli are combined with the procedure of Hübner et al. (2010). To test this hypothesis, we used the same stimuli and task as in Experiment 1, but applied the procedure as in Hübner et al. Specifically, stimuli were presented only for 165 ms, participants had to indicate their decision by pressing a corresponding key with their index or middle finger of their right hand, respectively, errors were signaled by a tone, and the RSI was 2000 ms. Moreover, whereas the flanking arrows had always been arranged vertically in White et al. (2011), we also included a condition with horizontally arranged items.

If the observed difference in fit performance between the DSTP and the SSP model was due to the applied stimulus type and task, then the SSP model should again be superior, at least for the condition with vertically arranged items. However, if the difference depended on other procedural differences between White et al.’s (2011) and Hübner et al.’s (2010) studies, then the DSTP model should now be better.

### Method

Fifteen participants (mean age 23 years, six male) with normal or corrected-to-normal vision, participated in the study. They were recruited at the Universität Konstanz and were paid 8 €/h. Apparatus and stimuli were the same as in Experiment 1, except that there was an additional stimulus condition with horizontally arranged flanker items (0.4°separation). The procedure was adopted from Hübner et al. (2010). Each trial started with a fixation cross of 400 ms, which was followed by a blank screen for 600 ms (cue-stimulus interval) and by a subsequent stimulus array presented for 165 ms. The pointing direction of the target arrow had to be indicated by pressing a corresponding key with the index or middle finger of the right hand, respectively. One second after the response, the next trial began (RSI = 2000 ms). Errors were signaled by short tone.

After two preliminary 16-trials practice blocks for each stimulus arrangement, the participants worked through 18 test blocks of 64 trials each in a 1 h session. Blocks with horizontally arranged items and those with vertically arranged items were presented in alternating order. Half of the participants started with a horizontal block, the other half with a vertical block. Responses faster than 200 ms or slower than 1500 ms were excluded from analysis (<0.3% of all data).

### Results and discussion

#### Mean performance

Latencies of correct responses were analyzed in a two-way ANOVA for repeated measurements on the factors *arrangement* (horizontal, or vertical), and *congruency* (congruent, or incongruent). The analysis revealed significant main effects of *arrangement*, *F*(1, 15) = 7.72, *p *< 0.05, and of *congruency*, *F*(1, 15) = 105, *p *< 0.001. Responses were slightly faster in the horizontal condition than in the vertical one (409 versus 417 ms). They were also faster for congruent than for incongruent stimuli (380 versus 446 ms). The RTs for the individual conditions are listed in Table 2. The mean ER was 8.38%. ERs were subjected to an ANOVA of the same type as for the RTs. The analysis revealed a significant main effect of *congruency*, *F*(1, 15) = 200, *p *< 0.001. It indicates that congruent stimuli produced a lower ER than incongruent ones (2.14 versus 14.6%). The ERs for the individual conditions are shown in Table 2.

The mean performance shows that the modified procedure produced similar congruency effects as in Experiment 1. Although responses to horizontally arranged arrows were reliably faster than those to vertically arranged ones (see Table 2), the congruency effects did not differ significantly between these stimulus types.

#### Model fits

The data of two participants had to be excluded from modeling, because they made no errors in at least one condition. The parameter and goodness-of-fit values obtained from fitting the models to the distributional data are shown in Table 3. If we consider the different values, then we see that this time the performance of the DSTP model was superior to that of the SSP model. This holds for both stimulus types and clearly demonstrates that the SPP model is not generally superior to the DSTP model. Moreover, our results indicate that arrow stimuli and the corresponding task were not responsible for the advantage of the SSP over the DSTP model in White et al.’s (2011) study and in our Experiment 1. Rather, they suggest that some procedural detail determines which model is superior.

## Experiment 3

Because Experiments 1 and 2 differed in several procedural details, it remained open which one was responsible for the reversal of model superiority. If we consider the mean performance between the two experiments, then it is obvious that the responses in Experiment 1 were slower and the variance of the RTs was larger (see Table 2). A possible reason for this pattern is the difference in stimulus duration. In Experiment 1, stimuli were displayed until response, whereas they were presented only for 165 ms in Experiment 2. The former setting may have encouraged participants to delay their response on some trials. To test whether stimulus duration was a crucial factor for model superiority, we applied in the present experiment the same procedure as in Experiment 2, except that the stimulus (vertically arranged arrows) now remained visible until response. If data obtained with a long stimulus duration are favorable for the SSP model, then this model should again be superior to the DSTP model as in the Experiment 1.

### Method

Sixteen participants (mean age 23.7 years, four male) with normal or corrected-to-normal vision, participated in the study. They were recruited at the Universität Konstanz and were paid 8 €/h. Apparatus and stimuli were the same as in the previous experiment, except that the arrows were always arranged vertically. Also the procedure was the same. This time, however, the stimuli remained visible until response. After a 48-trials practice block, participants worked through 16 test blocks in a 1.5 h session. Each test block consisted of 64 trials. Responses faster than 200 ms or slower than 1500 ms were excluded from data analysis (<0.2% of the data).

### Results and discussion

#### Mean performance

The latencies of correct responses were analyzed in a one-factor ANOVA for repeated measurements on the factor *congruency* (congruent, or incongruent). Responses were faster for congruent than for incongruent stimuli, *F*(1, 16) = 95.9, *p *< 0.001 (see Table 2). The mean ER was 4.76%. The ERs were subjected to an ANOVA of the same type as for the RTs. A significant effect of *congruency*, *F*(1, 16) = 47.3, *p *< 0.001, revealed higher ER for that incongruent than for congruent stimuli.

Although the mean performance shows again the usual congruency effects (see Table 2), compared to the vertical-stimulus condition in Experiment 2, they were smaller in the present experiment (RT: Δ 47 versus Δ 63 ms; ER: Δ 6.42 versus Δ 13.0%). Thus, it seems that the longer stimulus duration in the present experiment reduced the response conflict. In contrast, the SD of the RTs was similar as in the previous experiment, but smaller than in Experiment 1.

#### Model fits

The DSTP and the SSP model were fit to the distributional data with the same procedures as in the previous experiments. The obtained parameters and goodness-of-fit values are given in Table 3. As can be seen, the long stimulus duration impaired the goodness-of-fit for both models. *G*^2^ increased for the DSTP model from 31.6 (vertical condition in Experiment 1) to 79.3, and for the SSP model from 65.3 to 105, so that the DSTP model remained superior. This also holds with respect to the BICs. Thus, stimulus duration seems not to be critical for model superiority. Another candidate could be the RSI, because it was unusually short in White et al.’s (2011) study (and also in the present Experiment 1). Whether the RSI indeed plays a critical role was tested in the next experiment.

## Experiment 4

In this experiment we tested effects of the RSI on model superiority. A preliminary experiment in our lab revealed that a short RSI of 350 ms, as used by White et al. (2011), and in our Experiment 1, is only feasible in combination with a long stimulus duration. Therefore, we used the same procedure as in Experiment 3, except that the RSI was now reduced from 2000 to 350 ms. This modification had the advantage that Experiments 3 and 4 differed only with respect to this single factor. Accordingly, it was possible to compare the performance between these two experiments statistically.

### Method

Sixteen participants (mean age 21.5 years, four male) with normal or corrected-to-normal vision, participated in the study. They were recruited at the Universität Konstanz and were paid 8 €/h. Apparatus and stimuli were the same as in the previous experiment. Also the procedure was the same as in Experiment 3, except that the RSI was reduced to 350 ms. For this objective we had also to abandon the fixation cross. After a 48-trials practice block, the participants worked through 16 test blocks of 64 trials each in a 45 min session. Responses faster than 200 ms or slower than 1500 ms were excluded from data analysis (<0.9% of the data).

### Results and discussion

#### Mean performance

The latencies of correct responses were analyzed by a one-factor ANOVA for repeated measurements on the factor *congruency* (congruent, or incongruent). It revealed a significant effect of *congruency*, *F*(1, 16) = 58.8, *p *< 0.001. Responses were faster for congruent than for incongruent stimuli (Table 2). The mean ER was 3.39%. The ERs were subjected to an ANOVA of the same type as for the RTs. It revealed a significant effect of *congruency*, *F*(1, 16) = 36.6, *p *< 0.001, indicating that incongruent stimuli produced a higher ER than congruent ones (Table 2).

#### Comparison with experiment 3

To assess the effects of the difference in RSI between the present experiment and Experiment 3 (Experiment 3: long RSI of 2000 ms; Experiment 4: short RSI of 350 ms), we subjected the mean RTs and their SDs for correct responses, and the mean ERs to two-factor ANOVAs with within-participant factor *congruency* (congruent, or incongruent), and between-participants factor *RSI* (long, or short), respectively. We report only results involving the factor *RSI*. A significant main effect of *RSI* in RT, *F*(1, 30) = 6.35, *p *< 0.05, indicates faster responses for the long than for the short RSI (403 versus 441 ms). Moreover, there was a significant interaction between *RSI* and *congruency* in RT, *F*(1, 30) = 4.94, *p *< 0.05, as well as in ER, *F*(1, 30) = 13.0, *p *< 0.01. These interactions indicate that congruency effects were generally stronger for the long than for the short RSI. Finally, there was a significant main effect of *RSI* in the SD, *F*(1, 30) = 9.47, *p *< 0.01. The SDs were smaller for the long than for the short RSI (86.1 versus 98.7 ms).

The comparison between Experiments 3 and this experiment shows that a shorter RSI increased the mean of the RTs as well as their SD (see also Table 2). It is well known that RT and RSI are negatively correlated (e.g., Rabbitt, 1980). A further effect of the reduced RSI was that the congruency effects were smaller, which suggests that during a short RSI the attentional weights can be maintained more optimally across trials. If this was the case, then the congruency-sequence effect should also have varied with RSI. The congruency-sequence effect is thought to reflect the phenomenon that the congruency effect is larger after a congruent trial than after an incongruent one (Gratton et al., 1992).

To test whether the congruency-sequence effect was affected by RSI, we subjected the mean RTs for correct responses and the mean ERs (trials with an error on the previous-trial were excluded) to three-factor ANOVAs with the within-participant factors *congruency* (congruent, or incongruent), and *previous-trial congruency* (congruent, or incongruent), and the between-participants factor *RSI* (long, or short). In RT it revealed a significant three-way interaction between all factors, *F*(1, 30) = 6.96, *p *< 0.05. It indicates that the congruency effect was reduced to a lesser extent for the long RSI, i.e., from 51 ms after a congruent trial to 41 ms after an incongruent one, than for the short RSI, where the reduction was from 44 to 21 ms. However, a further analysis revealed that the effect was also significant for the long RSI, *F*(1, 15) = 28.1, *p *< 0.001. In ER there was a significant previous-trial congruency effect, *F*(1, 30) = 22.7, *p *< 0.001, which, however, was not modulated by RSI.

#### Model fits

The DSTP and the SSP model were fit to distributional data with the same procedures as in the previous experiments. One participant had to be excluded, because she made no errors in the congruent condition. Parameter values and goodness-of-fit measures are listed in Table 3. As can be seen, the short RSI had crucial effects on model superiority. Although the pure goodness-of-fit (*G^2^*) was still slightly better for the DSTP model, the model-selection criterion (BIC) was now again smaller for the SSP model. Thus, these results suggest that a short RSI is crucial for model selection. Its duration determines whether the SSP model or the DSTP model is superior.

## General Discussion

An important question is whether spatial selective attention improves discretely or continuously during stimulus processing and response selection in the flanker task. If one considers the corresponding studies, though, then the results are inconclusive. Whereas Hübner et al. (2010)found that the discrete DSTP model accounts better for flanker-task data than various continuous models, White et al. (2011)observed that their continuous SSP model was superior. Because both studies used different tasks and procedures, it was not possible to decide whether the superiority of the SSP model holds generally, or only under certain conditions. Therefore, in a first step, we also applied the SSP model to Hübner et al.’s data, and found that its fit was worse than that of the DSTP model (see Table 1). This shows that the SSP model is not generally superior, and suggested that methodological details were responsible for the opposite conclusions. To investigate which details are crucial in this respect, we conducted a series of experiments.

In our first experiment we tested whether White et al.’s (2011) results are replicable in our lab. The results show that the SSP is indeed superior to the DSTP model under the specific method applied in that study. Although the pure goodness-of-fit was slightly better for the DSTP model, the model-selection statistics (BIC) was in favor of the SSP model, due to the fewer parameters of that model. This result supported our hypothesis that specific procedural details were responsible for the incompatible results between Hübner et al.’s (2010) and White et al.’s (2011) study.

In our second experiment we examined the role of task and stimuli for model superiority. To this end, we used the same stimuli and task as White et al. (2011), but applied the experimental procedure of Hübner et al. (2010). It turned out that now the DSTP model was superior, which indicated that stimuli and task were not crucial for the superiority of the SSP model in White et al.’s (2011) study. To examine exactly which procedural details were essential in this respect, we ran two subsequent experiments in which we tested the role of stimulus duration (Experiment 3), and of RSI (Experiment 4). The results demonstrate that the RSI is the crucial variable. The SSP model seems to be superior to the DSTP model only if the RSI is short, as in Experiments 1 and 4, where the RSI was 350 ms, compared to the 2000 ms in Experiments 2 and 3.

**Figure 2:**
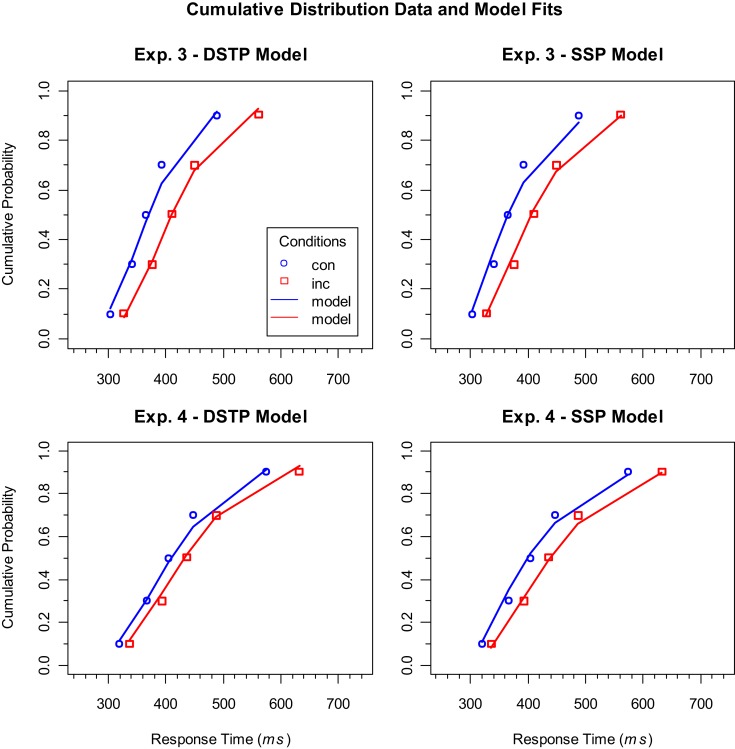
**Cumulative distribution functions of RTs for correct responses to congruent (con) and incongruent (inc) stimuli in Experiments 3 (upper panels) and 4 (lower panels)**. The symbols represent the vincentized data and the lines the corresponding model performance of the DSTP model (left panels) and SSP model (right panels).

The considerations so far do not answer the question of why the RSI actually had an effect on model superiority. Thus, to further examine why the RSI had such an effect on model superiority, it might be helpful to consider the distributional data in detail. We did this exemplarily for our last two experiments, because they differed only in RSI, and model superiority was reversed between them. Figure 2 shows the empirical cumulative distribution functions of the RTs for correct responses in the different conditions and the corresponding model fits. As can be seen, both models fit the data for both experiments relatively well, and differences are hardly noticeable by visual inspection. If we consider the corresponding goodness-of-fit values, i.e., to what extent the deviations for correct responses contributed to *G^2^*, then we find that the SSP model was superior to the DSTP model in the two experiments (Experiment 3: DSTP 58.8, SSP 48.5; Experiment 4: DSTP 49.7, SSP 42.7). This superiority, though, does not hold generally. In Experiment 1, for instance, the DSTP fit correct responses similarly good or even better than the SSP model (horizontal condition: DSTP 17.5, SSP 23.3; vertical condition: DSTP 27.3, SSP 27.3).

These considerations suggest that the main difference in model performance between Experiments 3 and 4 was mainly present in the error distributions, which had also been used for model fitting, because variations in accuracy are essential for the present objective. Indeed, if we consider the contributions of error data fitting to the goodness-of-fit statistics, then it is obvious that both models had more difficulties with fitting the data of Experiment 3, but also that this was much more the case for the SSP model (Experiment 3: DSTP 20.5, SSP 56.3; Experiment 4: DSTP−2.08, SSP 7.88). To demonstrate the meaning of these differences, we visualized the error data by plotting conditional accuracy functions (CAFs). For our objective these functions are more informative than cumulative RT distributions of errors, because they more directly show how accuracy (and selectivity) improved with RT (cf. Gratton et al., 1992).

**Figure 3:**
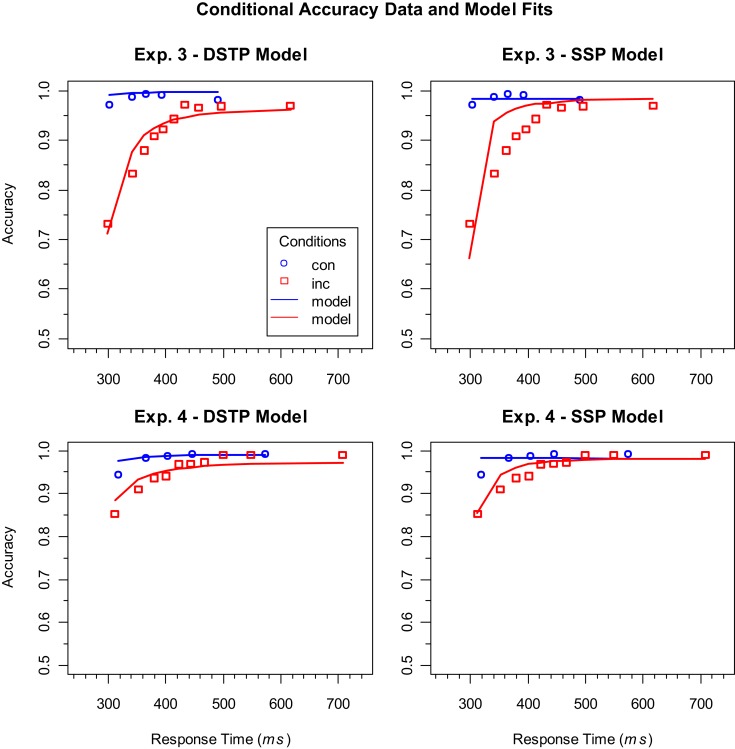
**Conditional accuracy functions (CAFs) for the congruent (con) and incongruent (inc) conditions in Experiments 3 (upper panels) and 4 (lower panels)**. The symbols represent the vincentized data and the lines the corresponding model performance of the DSTP model (left panels) and SSP model (right panels). Note that the models were fit to the corresponding cumulative RT distributions and not to the CAFs.

Figure 3 shows the empirical as well as the theoretical CAFs computed from the estimated model parameters. As can be seen, the empirical functions have the expected general form. Accuracy is relatively low for fast responses to incongruent stimuli, but then improves with RT and finally reaches a similarly high level as the CAFs for congruent stimuli. If we consider the upper two panels, which represent the data and model fits for the long RSI condition (Experiment 3), then we see why the DSTP model fits the data better than the SSP model. The SSP model underestimates the accuracy for very fast responses to incongruent stimuli and predicts that it improves too quickly. In other words, the predicted slope of the CAF for incongruent stimuli is too steep. In contrast, the DSTP model fits the increase in accuracy for the incongruent stimuli rather well. This shows that the SSP model does not always adequately fit the slope of steep CAFs. The CAFs for the short RSI condition (Experiment 4) can be seen in the lower panels of Figure 3. Obviously, accuracy for fast responses to incongruent stimuli was already relatively high under this condition, indicating a correspondingly high spatial selectivity. For these data the fit of SSP model was similarly good as that of the DSTP model.

In a recent article, White et al. (2012) compared the performance of their SSP model with that of two specific Bayesian observer models (Yu et al., 2009), which also assume a continuous-improvement of selectivity. They could show that the crucial difference of the observer models compared to other continuous-improvement models is not their Bayesian decision part, but the assumed attentional mechanisms, which were based on compatibility bias and spatial uncertainty. White et al. (2012) fit the observer models to data from White et al. (2011), and also considered CAFs. The comparison revealed that the SSP model was superior, mainly because it adequately captured the steep slopes in the CAFs for incongruent stimuli. However, as we have seen in the present study, predicting a steep slope might not always be appropriate. Accordingly, as the CAFs were less steep in the present study for conditions with a long RSI, one might ask whether the observer models might be superior to the SSP model in this case, or even better than the DSTP model. If we consider the fits of the different models to the data from Experiment 1 in White et al. (2011), then we see that the SSP model was slightly superior to the DSTP model $\left(\chi_{\text{SSP}}^{2}=632\;\text{vs}.\;\chi_{\text{DSTP}}^{2}=656\right),$ whereas both models largely outperformed the Bayesian observer models ($\chi_{\text{Observer}}^{2}=1553,$ and 4411, respectively; see White et al., 2012). Thus, given the moderate differences in performance between the DSTP and the SSP model in the present experiments, it is highly likely that the SSP model as well as the DSTP model remain superior to the observer models also for longer RSIs, as applied in the present study. In any case, we can conclude that there is no single continuous-improvement model, at least up to now, that is generally superior to the DSTP model.

Given our result that the congruency effects were relatively small in the experiments with a short RSI (Experiments 1 and 4), one could speculate that this is a necessary condition for the superiority of the SSP model. However, if we consider the congruency effects in White et al.’s (2011) study, then we see that they were also relatively large. Thus, it seems that the size of the congruency effect is not crucial. What could instead be responsible is the fact that a short RSI produces broader RT distributions, which is compatible with the observed greater SD of the RTs. As can be seen in Figure 2, slow responses were delayed to a larger extent than fast responses.

Thus, we have the result that the SSP model, which so far represents the most successful continuous-improvement mechanism of spatial selectivity, is not generally superior to the discrete improvement DSTP model, but only under specific conditions. One of these conditions, as figured out in the present study, is given if the RSI is rather short. In our experiments, RTs and their SD decreased with an increasing RSI, as can be seen by considering the distributions in Figure 2. This effect can have different origins. For instance, with a short RSI participants might not have been well prepared for responding again shortly after an executed response, presumably, because there is some refractory period (Rabbitt, 1969). There is also evidence that a short RSI delays the onset of sensory evidence accumulation (e.g., Seibold et al., 2011). Furthermore, sequential effects suggest that short RSIs generally increase automatic facilitation, whereas long RSIs increase the impact of expectations (e.g., Soetens et al., 1985).

Obviously, the short RSI in Experiments 1 and 4 reduced the congruency effects, suggesting that attentional adjustments could better be maintained from one trial to the next. This would be in line with other results showing that carry-over effects between trials depend on the RSI. Egner et al. (2010), for instance, varied the RSI from 500 to 5000 ms in steps of 500 ms. They found that congruency-sequence effects decreased with an increasing RSI, and were absent for RSIs longer than 2000 ms. Indeed, also in our data we found such a modulation. The comparison between Experiments 3 and 4 revealed that the congruency-sequence effect was larger for the short than for the long RSI, although it was still present for the long RSI. It should be noted, however, that in our experiments the congruency-sequence effect does not necessarily reflect some kind of conflict adaptation (Botvinick et al., 2001), because with the present set of stimuli there were unequal proportions of target/response repetitions in the different congruency-sequences, which might also have contributed to the observed sequential effects (Mayr et al., 2003).

Our results support the notion that the specific RSI in an experiment can have various positive and/or negative effects on performance. Choosing a long RSI is no guarantee that there are no sequential effects. However, they might be reduced, compared to short RSIs, at least with respect to the more automatic processes. If we consider our model parameters (Table 3), then they suggest that the RSI mainly affected early attentional selection, i.e., early spatial filtering. If we compare the corresponding values between Experiments 3 and 4 for the DSTP model, then we see that the partial rate for the target (μ_ta_) was only somewhat smaller under a short RSI (Experiment 4), whereas that for the flankers (μ_fl_) was substantially reduced. These values indicate that early selection was more effective under the short RSI. Similarly, for the SSP model the initial diameter (sd_a_) of the spotlight was smaller in Experiment 4, as was the perceptual evidence (*p*). Furthermore, for both models the response criterion (*A/B*) was higher for the short RSI.

Taken together, our study shows that, different from White et al.’s (2011) suggestion, the continuous SSP model is not generally superior to the discrete DSTP model, not even for explaining the performance in simple flanker tasks. Rather, it offers a more parsimonious description of flanker-task data only under specific conditions. One of such conditions, as also shown in the present study, is a relatively short RSI. Thus, it remains open whether selectivity of spatial attention improves continuously or discretely. As both models largely mimic each other, many comparisons under various conditions might probably be necessary to reach a final decision of which attentional mechanism is valid.

## Conflict of Interest Statement

The authors declare that the research was conducted in the absence of any commercial or financial relationships that could be construed as a potential conflict of interest.

## Footnotes

^1^Because we fit group data, the average number of valid trials per person was used as number of observation (*N*) for computing these values. For the present objective this is sufficient, because these measures merely served for comparing the models.
